# Effectiveness of Traditional Japanese Herbal (Kampo) Medicine, Daiobotanpito, in Combination with Antibiotic Therapy in the Treatment of Acute Diverticulitis: A Preliminary Study

**DOI:** 10.1155/2013/305414

**Published:** 2013-09-03

**Authors:** Keiko Ogawa, Koji Nishijima, Fumio Futagami, Takashi Nakamura, Genichi Nishimura

**Affiliations:** ^1^Clinic of Japanese-Oriental (Kampo) Medicine, Department of Otorhinolaryngology & Head and Neck Surgery, Kanazawa University Hospital, 13-1 Takaramachi, Kanazawa, Ishikawa 920-8641, Japan; ^2^Department of Surgery, Japanese Red Cross Kanazawa Hospital, Mima 2-251, Kanazawa 921-8162, Japan

## Abstract

In traditional Japanese herbal (Kampo) medicine, daiobotanpito (DBT) or Da Huang Mu Dan Tang in Chinese has been used in medical treatment of acute diverticulitis for many years based on the experience. Our aim was to investigate whether the treatment of acute diverticulitis can be treated with intravenous antibiotics plus orally administrated DBT than intravenous antibiotics alone. A retrospective nonrandomized open-label trial was established to compare patients with acute diverticulitis who received oral DBT associated with intravenous antibiotics with those who received intravenous antibiotic alone. We included 34 patients, eleven patients in group 1 with DBT and 23 patients in group 2 without DBT. Both groups were comparable in patient demographics and clinical characteristics. There was a significantly better outcome in the group treated with DBT than in the group without DBT when comparing duration of fever, abdominal pain, and antibiotics administration. A trend toward a day shorter mean hospital stay and fasting was seen in group 1, although this did not reach statistical significance. In conclusion, most patients with acute diverticulitis can be managed safely with oral DBT. Although randomized, double-blind study must be done, we could show the possibility to use daiobotanpito as an additional option in treating acute diverticulitis.

## 1. Introduction

The prevalence of diverticular disease is estimated to range between 20% and 60% in the general population [1, 2]. The most frequent complication of diverticular disease is acute diverticulitis. Although 75% of patients with diverticulosis remain symptom-free during their lifetime, the prevalence of patients that require medical or surgical treatment has increased 16% in the last 20 years, consequently increasing morbidity [3–5]. 

According to the guideline of the American Society of Colon and Rectal Surgeons [6], the initial treatment of uncomplicated colonic acute diverticulitis is bowel rest and broad-spectrum antibiotics. Patients who do not respond to medical therapy should be considered for surgery depending on the clinical situation. Selected abscess detected on abdominal ultrasound may be drained percutaneously. Elective resection is recommended after two well-documented attacks, depending on age, medical fitness of the patient, and severity of the attack. Complicated diverticulitis is diverticulitis associated with obstruction, stricture, fistula, or abscess. In cases of perforated diverticulitis, after a careful selection of patients, the technique of choice should be resection with primary anastomosis.

The unique role played by traditional Japanese herbal (Kampo) medicine is gradually attracting worldwide attention. In Kampo medicine, daiobotanpito (DBT; Da-Huang-Mu-Dan-Tang) is used in relatively strong patients with Yang excess in the interior layers of the body with signs of local Qi congestion and blood stasis (Oketsu). Traditionally, it has thus been used for abscesses of the intestine, such as diverticulitis or appendicitis. From the view of Kampo medicine, DBT drains heat, breaks up blood stasis, disperses clumping, and reduces swelling. It is especially used in relatively strong patients with abdominal distention and constipation. Daiobotanpito consists of five crude drugs: *Rhei Rhizoma*, *Natrium sulfuricum*, *Moutan Cortex*, *Persicae Semen*, and *Benincasae Semen*. For example, *Rhei Radix et Rhizoma* and *Moutan Cortex* drain heat and dispel blood stasis. They are attributed to heat and constraint that allows the formation of toxins which secondarily leads to the formation of pus. The appropriate strategy of Kampo medicine is to discharge the toxin, eliminate the phlegm, clear the heat, and open the restraint [7].

As described above, surgery should be performed in patients who do not respond to medical therapy in difficult or complicated clinical situation. DBT may be an alternative or supportive therapy in acute diverticulitis to avoid surgical treatment. Even though DBT is traditionally used for diverticulitis, no clinical reports or trials have been published in the English literature.

The authors hypothesize that additional oral DBT is superior to the standard therapy in acute diverticulitis. This paper aims to investigate the effectiveness of the treatment of acute colonic diverticulitis with Kampo medicine, DBT.

## 2. Patients and Methods

### 2.1. Patients

Between April and November 2012, standard treatment was offered to 34 patients who were diagnosed to have acute diverticulitis by surgeons in the Japanese Red Cross Kanazawa Hospital. 

Diagnosis was based on the image of computed tomography (CT), diverticula-like structure in accordance with tenderness or abdominal pain, a thickening of colon wall, signs of inflammation of the pericolonic fat, the tissue density, and vascular involvement. Pericolonic abscess, free air or extravasation, and accumulation of fluid were also noticed to predict prognosis.The standard treatment for acute uncomplicated diverticulitis is bowel rest (fasting), intravenous fluids, and intravenous antibiotics. Antibiotics were continued until CRP becomes negative. 

Eleven of 34 patients agreed to add Kampo treatment, DBT. We used daiobotanpito extract which consists of five crude drugs in fixed proportions: *Rhei Rhizoma* (2.0 g), *Natrium sulfuricum *(1.8 g), *Moutan Cortex* (4.0 g), *Persicae Semen* (4.0 g), and *Benincasae Semen *(6.0 g), in 7.5 g of extract. We excluded 4 patients who received an operation from the last analysis. Therefore, we evaluated ten patients treated with DBT and 20 patients treated with only antibiotics. We checked the patients' background (age, sex, and location, etc.) (Table 1) to demonstrate that both groups were almost homogenous. Although there were significant differences in number of episodes of diverticulitis and previous diverticulitis, there was no significant difference in other factors.

### 2.2. Study Design

An open-label nonrandomized retrospective controlled trial was designed to investigate the efficacy of the standard therapy versus the standard therapy plus DBT for acute diverticulitis. The study protocol was designedin accordance with the ethical principles in the Declaration of Helsinki and regional regulations.

No restrictions were imposed on the standard treatments for acute diverticulitis or any other disease, while DBT was administered. If the clinical evolution was right, a regular diet was initiated, and the patient was discharged and was controlled as an outpatient a week later.

There were two treatment groups. (1) “DBT group” (group 1) patients began antibiotics and DBT administration within 24 h after admission when their symptoms improved and CRP became negative. After obtaining informed consent, DBT extract (Tsumura, Tokyo, Japan) at 2.5 g was administered three times a day, 7.5 g per day. (2) The “Without DBT group” (group 2) patients only received antibiotics intravenously. 

Clinical improvement was defined as pain decrease, absence of abdominal pain or tenderness, absence of fever, and negative CRP.

The endpoint of the study was duration of fever, abdominal pain, antibiotics administration, days of initiation of regular diet, and days of hospital stay.

### 2.3. Statistical Analysis

The data were analyzed using Excel; 2010. Analysis was restricted to patients with diverticulitis. Continuous variables were analyzed with Student's *t*-test examining the endpoints febrile days, days with abdominal pain, days of hospital stay, days of antibiotic administration, and days to initiation of regular diet. A *P* value of less than 0.05 was considered statistically significant.

## 3. Results

Of the 34 patients, four were excluded because of the operation. Clinical data were thus investigated for 31 patients, 10 from group 1 and 20 from group 2. There were no adverse effects of DBT in group 1.

Both groups were comparable in patient demographics and clinical characteristics. Considering all the patients, 5 (14.7%) had suffered previous episodes of diverticulitis (mean of episodes 1.4). Data on both groups are shown in Table 1. Temperature on admission was below 38°C in 20 patients (58.8%).

In all cases, CT scan showed bowel wall thickness with different degrees of pericolic fat infiltration and the presence of diverticula. The two groups were comparable with respect to age, sex, previous diagnosis of diverticulosis, previous episodes of diverticulitis, duration of symptoms before admission, and CRP/WBC at admission (Table 1).

All patients in group 1 except two were discharged before day 9 of admission, and all patients in group 2 were discharged before day 15. Data of clinical evolution of both groups are shown in Table 2.

There was a significantly better outcome in the groups treated with DBT than in the group without DBT when comparing for duration of fever, abdominal pain, and antibiotics administration. A trend toward a day shorter mean hospital stay and fasting was seen in group 1, although this did not reach statistical significance.

There was no adverse effect with antibiotics and DBT in both groups. There was complete resolution of symptoms in both groups.

## 4. Discussion

The main reason that led us to propose this study was the necessity to prove the real effect of DBT in acute diverticulitis. 

In this study, we showed the significantly better differences in the groups treated with DBT combined with antibiotics than in the group without DBT when comparing for duration of fever, abdominal pain, antibiotics administration, and days of initiation of regular diet. A trend toward a day shorter mean hospital stay and fasting was seen in group 1, although this did not reach statistical significance. Although there were more patients with episodes of recurrent diverticulitis in group 1 than in group 2, there were some advantages in the standard therapy plus DBT, which may improve patients' quality of life (QOL). Operation might be prevented, even in cases with recurrent diverticulitis. This also implies an economical advantage.

Diverticula can occur at any sites in the colon; however, due to the thickened consistency of the stools, diverticulitis mostly occurs in the sigmoid colon. The standard treatment for acute uncomplicated diverticulitis has been bowel rest (fasting), intravenous fluids, and intravenous antibiotics. Case-by-case therapy is initiated by the attending surgeon, so there was a diversity of the antibiotics such as ceftriaxone, flomoxef, sulbactam, cefmetazole, and doripenem, used for patients in this study. This is caused by variability in the use of antibiotics in clinical practice among centers in the management of acute diverticulitis particularly relating to the selection of antibiotic. Additionally, while there are a lot of trials focused on surgical treatment, there are very few studies dedicated to medical treatment of acute diverticulitis. A review of the published data confirmed the impression that there is no standardization in the medical treatment of uncomplicated acute diverticulitis [8]. In the next research, randomization and unified antibiotic regimen would be necessary to investigate the effect of DBT. Treatment with DBT is perhaps to be considered as an effective option for the treatment of acute uncomplicated colonic diverticulitis. Also the findings indicate that this treatment may be suitable both for first occurrence and, importantly, for recurrentoccurrences of the disease although we could not find clinical report even from the Japanese literature.

In Kampo medicine, internal abscesses generally mean abscesses of the lungs and intestines. Similar to external abscesses, they are attributed to heat and constraint that allows the formation of toxins. This secondarily leads to the formation of pus. The appropriate strategy is to discharge the toxin, eliminate the phlegm, clear the heat, and open the constraint. If there is also clumping of heat with stasis and stagnation, herbs such as *Rhei Radix et Rhizoma* (Da Huang *大黄*) and *Moutan Cortex* (Mu Dan Pi *牡丹皮*) can be added to drain heat and dispel stasis [7]. In this concept, DBT is one of the most suitable formulas for treatment of acute diverticulitis.

DBT might have preventive effects on the recurrence of diverticulitis, although prognosis of patients was not investigated in this study. 

Most herbal medicines are orally administrated, and most components of these medicines are inevitably brought into contact with the intestinal microflora in the alimentary tract. Some are transformed by the intestinal bacteria before their absorption from the gastrointestinal tract [9]. Although administration of intravenous antibiotics may influence the intestinal microflora and the effect of DBT might be changed compared with classical therapy, this study showed the possibility to use both intravenous antibiotics and DBT at the same time.

Daiokanzoto (DKT), a Kampo medicine that includes the combination of two crude drugs, rhubarb and glycyrrhizae, is clinically effective for constipation. The combination of two crude drugs, rhubarb and glycyrrhize radix, are also contained in DBT. Matusi et al. showed the influence of glycyrrhizae radix and antibiotics on the purgative action of sennoside A of rhubarb from DKT in mice [10]. The purgative actions of rhubarb and sennoside A were significantly intensified when glycyrrhizae radix was coadministered orally to mice. On the other hand, the purgative action of sennoside A was significantly reduced by the preadministration of minocycline (tetracyclin antibiotics), whereas that of DKT was not affected. Other crude drugs such as glycyrrhizae radix may have the ability to recover the action of other drugs suppressed by antibiotics via an unknown mechanism. This is the advantage of complexity of Kampo medicine.

DBT consists of five crude drugs: *Rhei Rhizoma*,* Natrium sulfuricum, Moutan Cortex, Persicae Semen, *and *Benincasae Semen*. Of these, *Rhei Rhizoma* or *rhubarb* are one of the most important traditional herbal medicines widely used in Kampo and traditional Chinese medicines for thousands of years, especially as a purgative. It might prevent bowel retention, overgrowth of toxic bacteria in patients with acute diverticulitis, and result in resolution of acute inflammation. 

Studies on other functions of rhubarb in modern medical research both in clinical and basic science settings have revealed that rhubarb has multiple effects including defervescence, anti-inflammatory actions, and especially expelling a variety of harmful materials such as endogenous as well as exogenous toxins from the bowel and the body.

It is shown that rhubarb protects against acute lung injury induced by LPS andthat rhubarb administration improves respiratory function of the body. Its effect is related to the regulation of the production of nitric oxide as well as phospholipase A andplatelet-activating factor activities [11]. These effects may be related to the improvement of inflammation in diverticulitis. 

As for *Moutan Cortex (MC)*, the root cortex of *Paeonia suffruticosa*, is a herbal medicine widely used as an analgesic, an antispasmodic, and an anti-inflammatory agent. *Moutan Cortex* is reported to inhibit the secretions of interleukin- (IL-) 8, a major mediator of acute neutrophil-mediated inflammation, and macrophage chemoattractant protein- (MCP-) 1, a potent mediator of chronic macrophage-mediated inflammation in human monocytic U937 cells [12].Recently, MC was shown to protect against sepsis induced by lipopolysaccharide/D-galactosamine [13]. The known chemical components of MC include paeonol, paeonoside, paeonolide, paeoniflorin, benzoylpaeoniflorin, oxypaeoniflorin, benzoyloxypaeoniflorin, and apiopaeonoside.

MC inhibited the LPS/r lFN-*γ*-induced expression of inducible nitric oxide synthase (iNOS) and TNF-*α* release. The LPS/r lFN-*γ*-induced activation of NF-*κ*B wasalmost completely blocked by MC [13]. 

*Persicae Semen* or *Prunus persica* is also well known as a traditional medicine in Japan, China, and other Asian countries. They are frequently used as an ingredient in a variety of Kampo and Chinese medicine formula, particularly those used to treat women's diseases. The chemical constituents of the herb include the cyanogenic glycosides, amygdalin, and prunasin as major components, along with glycerides, sterols, and emulsion. Amygdalin is also abundant in the seeds of bitter almond and apricots of the *Prunus* genus and other rosaceous plants. *Persicae Semen* have antiedema, antiwrithing, and anti-inflammation activities.

Each of these crude drugs has some anti-inflammatory effects, and the combination of these crude drugs may produce the synergy of Kampo medicine.

## 5. Conclusion

Most patients with acute diverticulitis could be managed safely with intravenous antibiotics plus oral DBT. Although randomized, double-blind study must be done, the patients benefit from the use of DBT as an additional option in the treatment of acute diverticulitis. Prognosis has to be investigated in the next study.

## Figures and Tables

**Table 1:** Characteristic of patients.

	Group 1 (*n* = 11)	Group 2 (*n* = 23)	*P* value
Age (mean)	25–77 (43.5)	25–79 (45.8)	0.34^a^
Sex (male/female)	1/10	5/18	0.365^b^
CRP	6.18	6.49	0.451^a^
WBC	10740	11408	0.266^a^
Previous diverticulitis	4 (36.3%)	1 (4.3%)	0.0137^b^
Number episodes of diverticulitis	0.45 (0–3)	0.043 (0-1)	0.00494^a^
Lesion (right/left)	9/2	19/4	0.955^b^
Operation	1 (9.1%)	3 (13.0%)	0.827^b^
BMI	21.6	23.3	0.082^b^

^a^Student's *t*-test.

^b^Fisher's exact test.

**Table 2:** Clinical evolution during admission.

Days	Group 1 (*n* = 10)	Group 2 (*n* = 20)	*P*
Start of oral diet	5.1	6.1	0.055
Antibiotic therapy	5.1	7.0	<0.05
Hospital stay	7.6	9.0	0.061
Abdominal pain	4.8	5.8	<0.05
Febrile	2.3	3.4	<0.05
